# Vermittlung kommunikativer Kompetenzen in der Lehre der HNO-Heilkunde: Vergleich zwischen einer Online- und Präsenzveranstaltung

**DOI:** 10.1007/s00106-024-01475-2

**Published:** 2024-04-10

**Authors:** Deniz Gür, Christian Offergeld, Götz Fabry, Alexander Wünsch

**Affiliations:** 1 Klinik für Psychosomatische Medizin und Psychotherapie, Medizinische Fakultät, Universitätsklinikum Freiburg, Albert-Ludwigs-Universität Freiburg, Hauptstraße 8, 79104 Freiburg im Breisgau, Deutschland; 2 Klinik für Hals-Nasen-Ohrenheilkunde, Medizinische Fakultät, Universitätsklinikum Freiburg, Albert-Ludwigs-Universität Freiburg, Freiburg, Deutschland; 3 Medizinische Psychologie, Medizinische Fakultät, Albert-Ludwigs-Universität Freiburg, Freiburg, Deutschland; 4 Medizinische Onkologie, Psychoonkologischer Dienst, Inselspital, Universitätsspital Bern, Universität Bern, Bern, Schweiz

**Keywords:** Videokonferenz, Medizinstudierende, Digitale Technologien, Unterricht, Simulationstraining, Videoconferencing, Medical students, Digital technology, Teaching, Simulation training

## Abstract

**Hintergrund:**

Kommunikative Kompetenzen gehören zu den wichtigsten Schlüsselqualifikationen der ärztlichen Tätigkeit. Inwieweit diese im medizinischen Unterricht der Hals‑, Nasen- und Ohrenheilkunde auch online erworben werden können, wird in dieser Studie untersucht.

**Fragestellung:**

Ein freiwilliges Online-Training zur Vermittlung kommunikativer Fertigkeiten wurde mit einem entsprechenden Präsenzformat verglichen. Dabei wurde der Frage nachgegangen, inwieweit sich Akzeptanz der beiden Formate sowie die Selbsteinschätzung der Studierenden hinsichtlich ihrer kommunikativen Fertigkeiten unterscheiden.

**Material und Methoden:**

Im Online-Training wurden die Studierenden über ein Video asynchron auf die Thematik vorbereitet. Danach konnten sie Gespräche mit Simulationspatient*innen online und synchron führen. Die Präsenzveranstaltung war in Aufbau und Dauer vergleichbar und fand in einem früheren Semester statt. Die Akzeptanz der jeweiligen Seminare wurde mit einem Fragebogen mit 19 Items erhoben, dabei wurde eine 5‑stufige Likert-Skala verwendet. Die Selbsteinschätzung der Kommunikationsfertigkeiten wurde über eine 10 cm lange visuelle Analogskala prä/post mit 16 Items erfasst.

**Ergebnisse:**

Beide Formate erreichten eine hohe Akzeptanz mit einer Durchschnittsnote (M) von 2,08 (Standardabweichung, SD: 0,54) für das Online-Format und M = 1,97 (SD = 0,48) für die Präsenzveranstaltung. Die Selbsteinschätzungen der Studierenden zu kommunikativen Fertigkeiten haben in der Online-Gruppe (M = 1,54, SD = 0,94) einen doppelten Zuwachs gegenüber der Präsenzgruppe gezeigt (M = 0,75, SD = 0,87).

**Schlussfolgerung:**

Diese Studie zeigt, dass die Vermittlung von kommunikativen Kompetenzen im Online-Format gut angenommen wurde und zu signifikanten Veränderungen in der Selbsteinschätzung der Kommunikationsfertigkeiten der Studierenden führte.

**Zusatzmaterial online:**

Die Online-Version dieses Beitrags (10.1007/s00106-024-01475-2) enthält Tab. S1.

## Abstract (en)

**Background:**

Communication skills are among the most important key qualifications of the medical profession. To what extent these can also be acquired online in medical education in otolaryngology is investigated in this study.

**Objective:**

A voluntary online training for the teaching of communication skills was compared with a corresponding face-to-face format. The question of the extent to which acceptance of the two formats and students’ self-assessment of their communicative skills differed was investigated.

**Materials and methods:**

In the online training, students were prepared for the topic asynchronously via a video. Thereafter, they were able to conduct consultations with simulation patients online and synchronously. The face-to-face training was comparable in structure and duration and took place in an earlier semester. The acceptance of both seminars was assessed by a questionnaire with 19 items on a five-point Likert scale. Self-assessment of communication skills was measured by a 10-cm visual analog scale pre/post with 16 items.

**Results:**

Both formats achieved high acceptance with an average score (M) of 2.08 (standard deviation, SD = 0.54) for the online format and M = 1.97 (SD = 0.48) for the face-to-face event. Students’ self-assessments of communication skills showed a twofold increase in the online group (M = 1.54, SD = 0.94) compared to the face-to-face group (M = 0.75, SD = 0.87).

**Conclusion:**

This study shows that teaching communication skills in the online format was well accepted and resulted in significant changes in students’ self-assessment of communication skills.

Die Pandemie machte es notwendig, ärztliche Kernkompetenzen im Medizinstudium online zu unterrichten. Inwieweit auch die Vermittlung von kommunikativen Fertigkeiten in der HNO-Heilkunde online möglich sein könnte, wird in dieser Studie nachgegangen. Dabei wurde ein kompaktes Kommunikationstraining online konzipiert und mit einem Präsenztraining, das in Aufbau und Inhalt vergleichbar war, gegenübergestellt. Das Online-Training erwies sich als machbar mit guter Akzeptanz und signifikanten Veränderungen in der Selbsteinschätzung der Studierenden.

## Relevanz

Kommunikative Kompetenzen gehören in allen Bereichen der Medizin zu den wichtigsten Schlüsselqualifikationen für die ärztliche Tätigkeit und können gelehrt und erlernt werden [1, 2]. In vielen medizinische Fakultäten bilden Kommunikationstrainings mittlerweile einen festen Bestandteil des Curriculums [2, 3]. Meist werden mit Simulationspatient:innen (SP) relevante kommunikative Aufgaben eingeübt, und es wird Studierenden die Möglichkeit gegeben, über gezieltes Feedback ihre kommunikativen Kompetenzen zu verbessern [4–6]. Nach derzeitigem Wissensstand wird empfohlen, die Vermittlung kommunikativer Kompetenzen zum einen longitudinal und zum anderen in Verbindung mit klinischen Inhalten zu unterrichten [7–10].

Vor diesem Hintergrund wurde eine interdisziplinäre Lehrveranstaltung im Rahmen des HNO-Blockpraktikums an der Medizinischen Fakultät der Albert-Ludwigs-Universität Freiburg entwickelt, welche die kommunikativen Kompetenzen der Studierenden in diesem Bereich fördern soll. Basiskompetenzen in der ärztlichen Gesprächsführung wurden in der Medizinpsychologie, Psychosomatik und in der Psychiatrie unterrichtet. Nun wurde eine Lehreinheit in Verbindung mit den klinischen Inhalten der HNO-Heilkunde konzipiert. Es bestand aus einer kurzen theoretischen Einführung und einem anschließenden Kommunikationstraining mit SP und einem fokussierten Feedback durch Beobachtende, SP und Tutor.

Aufgrund der Coronapandemie musste das bestehende Präsenzformat im Jahr 2020 kurzfristig in ein Online-Format adaptiert werden. Dieser Herausforderung konnte die Univ.-HNO-Klinik Freiburg mit einem kompletten E‑Curriculum in kürzester Zeit erfolgreich begegnen [11]. Aus anderen Studien geht hervor, dass die Online-Form eine hohe Akzeptanz unter Studierende gefunden hat sowie einen hohen Lerneffekt erzielen kann [12–17]. Ob dies auch in dem konzipierten Online-Format nachgewiesen werden kann, soll in dieser Studie untersucht werden. Die Fragestellungen lauten:

1. Wie wird eine Online-Veranstaltung im Vergleich zu einer Präsenzveranstaltung evaluiert?
2. Wie unterscheidet sich die Selbsteinschätzung der Studierenden bei einer Online-Veranstaltung im Vergleich zu einer Präsenzveranstaltung?

## Material und Methoden

### Aufbau der Seminare (Interventionen)

Das Online-Format war in Inhalt und Aufbau an die Präsenzveranstaltung gleich gestaltet. Die Präsenzveranstaltung war jedoch verpflichtender Teil des HNO-Blockpraktikums, die Online-Veranstaltung freiwillig. Die theoretische Hinführung aus der Präsenzveranstaltung wurde in ein Video zu „kommunikativen Kompetenzen“ **(**Tab. 1**)** und „Audiologie“ überführt, den die Studierende asynchron vor dem Online-Training ansehen konnten. Darin wurden u. a. Ursachen für Hörstörungen, Indikationen für ein Cochleaimplantat und Interpretationen von Sprachaudiogrammen vermittelt. Diese Videos wurden auf einer zentralen Lernplattform hochgeladen.

**Table 1:** 

Kontaktaufnahme (winken)
Störlärm vermeiden
Blickkontakt halten
Beleuchtung ggf. anpassen
Gesprächsthema klar benennen
Ankündigung von Fragen
Deutliches Mundbild
Langsam sprechen
Pausen machen
Nicht schreien

Die anschließende Trainingseinheit bestand aus einer Kleingruppenarbeit mit konkreten Übungsmöglichkeiten. In der Online-Version wurden die Studierenden über eine Plattform für Videokonferenzen in „break-out rooms“ zugeteilt. Jeder Kleingruppe war ein SP und ein*e Tutor*in zugeordnet. Pro Kleingruppe hatten 2 Studierende die Möglichkeit, eine Befundbesprechung bei einem durch einen SP dargestellten hörbehinderten Menschen durchzuführen. Dabei wurde der Fall einer Patientin gewählt, die einen Hörsturz erlitten hatte und der nun ein Cochleaimplantat empfohlen wird.

Die anderen Studierenden erhielten gezielte Beobachteraufgaben, zu denen sie dann dem übenden Studierenden ein Feedback geben sollten. Die Beobachtungsaufgaben basieren auf der COM-ON-Check Rating Scale, die wiederum theoretisch auf dem SPIKES-Modell zur Übermittlung schlechter Nachrichten aufgebaut ist [18, 19]. Die 6 Schritte des SPIKES-Modells sind: Setting, Perception, Invitation, Knowledge, und Strategy and Summary. Zudem wurde das NURSE-Modell von Back et al. [20] in die Beobachteraufgaben integriert, das Empfehlungen für den Umgang mit Emotionen im Gespräch gibt. Das Akronym NURSE steht für: Naming (offene Benennung der Emotionen), Understanding (Äußerung von Verständnis für die Gefühle), Respecting (Anerkennen und Respektieren der Emotionen der Patient*innen), Supporting (Bewältigungstechniken der Patient*innen würdigen und Bereitschaft zur Hilfe ausdrücken) und Exploring (gezielte Nachfragen, um Empathie und Interesse zu zeigen und Hinweise auf die Gefühle der Patient*innen zu bekommen). Nach dem Gespräch von etwa 15 min schloss sich zunächst eine Selbstreflexion der/des übenden Studierenden an, gefolgt vom Feedback der Beobachtenden mit anschließendem Feedback des*der SP und des*der Tutor*in (Tab. 2).

**Table 2:** 

*Anamnese – etwa 6* *min*	Gezielte Abfrage zu den spezifischen Informationen der HNO-Anamnese zur Diagnosestellung und zum Führen des späteren Beratungsgesprächs
*Befundbesprechung – etwa 4* *min*	Erklären des aktuellen Hörtests in kurzen Worten
*Beratungsgespräch – etwa 5* *min*	Aufklärung des*der Patienten*in über die Diagnose
	Erläutern der Ätiologie des vorliegenden Krankheitsbildes
	Beraten bezüglich der notwendigen Diagnostik und zu erwartender Behandlungsstrategien
*Feedbackrunde – etwa 15* *min*	Studierende in der Arztrolle: Selbstreflexion
	Teilnehmende: Feedback anhand spezifischer Beobachteraufgaben
	Simulationspatient*in
	Tutor*in

### Datenerfassung

Es wurden Fragebögen zur Evaluation, zur Selbsteinschätzung und soziodemografische Daten erfasst und über einen Pseudonymisierungs-Code zugeordnet. Im Online-Kurs wurden die Fragebögen über ein Online-Programm erfasst.

Im Fragebogen zur Evaluation wurden seminarbezogene Aspekte und einzelne Bausteine der Seminare bewertet mit Antwortmöglichkeiten auf einer Likert-Skala zwischen 1 „trifft voll zu“ und 5 „trifft gar nicht zu“. Darüber hinaus gab es die Möglichkeit für einen Freitext.

Für die Selbsteinschätzung zur kommunikativen Kompetenz wurde eine visuelle Analogskala (VAS) mit 10 cm eingesetzt mit den Polen „sehr sicher“ und „sehr unsicher“, „sehr gut“ und „sehr schlecht“ oder „sehr leicht“ und „sehr schwer“. Die VAS wurden vor und nach dem Seminar ausgefüllt. Dabei wurden relevante Inhalte im Bereich der kommunikativen Kompetenz erfasst, wie z. B. die Strukturierung des Gesprächs, das Verwenden einer deutlichen Sprache oder das Halten des Blickkontakts.

### Rekrutierung

Das Seminar „Kommunikative Kompetenzen in der HNO“ erfolgte als verpflichtender Teil für die Studienkohorte von etwa 160 Studierenden während des Sommersemesters 2019 und im Wintersemester 2019/2020, die für das HNO-Blockpraktikum angemeldet waren. Das Ausfüllen der Begleitforschung war freiwillig. Im Wintersemester 2020/2021 und Sommersemester 2021 wurden allen Studierenden, die für das HNO-Blockpraktikum angemeldet waren, über das Angebot des Online-Kurses informiert. Die Teilnahme am Online-Seminar war freiwillig.

### Auswertung der Daten

Die statistische Analyse wurde mittels IBM® SPSS® Statistics 21 (Fa. IBM Corp., Armonk, NY, USA). durchgeführt. Fehlende Daten wurden anhand eines χ^2^-Tests auf Zufälligkeit geprüft. Da diese unter 5 % lagen, wurden die restlichen einzelnen Fehlwerte durch Mittelwerte ersetzt, um einen noch kleineren Datensatz zu vermeiden. Zur Evaluation der Seminare wurden Mittelwerte und Standardabweichung (SD) mithilfe des t‑Tests für unabhängige Stichproben mit einem Konfidenzintervall von 95 % mit Daten der Präsenzveranstaltung verglichen. Für die Auswertung der kommunikativen Kompetenzen, erfasst über die VAS, wurde eine Varianzanalyse mit Messwiederholung (ANOVA) gerechnet Dabei wurden sowohl die Innersubjekt- als auch die Zwischensubjekteffekte interpretiert.

## Ergebnisse

### Stichprobenbeschreibung

Das Teilnehmerkollektiv in der Präsenzgruppe waren 108 und in der Online-Gruppe 29. Weitere Details finden sich in Tab. 3.

**Table 3:** 

	Präsenzkurs	Online-Kurs
*Anzahl n*	108	29
*Alter, Jahre, M (SD)*	24,4 (4,4)	24,1 (2,7)
*Geschlecht*		
*Männlich ,n (%)*	33 (30,6 %)	11 (37,9 %)
*Weiblich, n (%)*	75 (69,4 %)	15 (51,7 %)
*Divers*	–	3 (10,3 %)
*Klinisches Semester*		
*≤* *3*	75 %	58,6 %
*≥* *4*	25 %	41,4 %

*n* Zahl der Probanden, *M* Mittelwert, *SD* Standardabweichung

### Evaluation des Trainings

Beide Lehrveranstaltungen wurden insgesamt gut bewertet mit einer Durchschnittsnote von 2,08 (SD = 0,54) für das Online-Format und 1,97 (SD = 0,48) für die Präsenzveranstaltung. Die statistische Überprüfung liefert keinen signifikanten Unterschied dieser Mittelwerte (t [129] = −1,11; *p* =0,269); (Tab. 4).

**Table 4:** 

		*n*	M	SD	*p*
**A. Seminarbezogene Aspekte**					
*Interesse am Thema*	Präsenz Online	102 29	1,88 1,83	0,88 0,97	0,773
*Aufbereitung der Themen*	Präsenz Online	102 29	1,74 2,03	0,84 0,87	0,096
*Anregung zur Auseinandersetzung mit dem Thema*	Präsenz Online	102 29	2,06 2,10	0,95 0,94	0,824
*Praxisrelevanz der Lehrinhalte*	Präsenz Online	102 29	1,3 1,28	0,64 0,53	0,830
*Praxisnahe Vermittlung der Lehrinhalte*	Präsenz Online	102 29	1,39 1,59	0,73 0,87	0,230
*Praktische Umsetzung inkl. Feedback*	Präsenz Online	102 29	1,69 1,86	0,91 1,13	0,387
KKT zum Umgang mit Patient*innen mit Kommunikationsdefiziten	Präsenz Online	102 29	1,25 1,28	0,70 0,59	0,829
*Besuch der Lehrveranstaltung*	Präsenz Online	102 29	1,61 1,76	0,94 1,12	0,466
**B. Einzelne Bausteine der Seminare**					
*Theoretische Einführung*	Präsenz Online	102 29	2,01 2,07	0,83 0,70	0,726
*Praktisches Üben mit SP*	Präsenz Online	102 29	1,44 1,69	0,80 1,14	0,185
*Beobachtungsaufgaben*	Präsenz Online	102 29	1,86 2,07	0,84 0,92	0,265
Feedback durch Kommiliton*innen	Präsenz Online	102 29	1,71 1,96	0,90 0,91	0,186
Feedback durch Schauspielpatient*in	Präsenz Online	102 29	1,44 1,55	0,73 0,83	0,484
Feedback durch Trainer*in	Präsenz Online	102 29	1,45 1,55	0,74 0,69	0,508
*Persönlicher Feedback-Bogen*	Präsenz Online	102 29	2,24 2,29	0,98 0,91	0,828
**C. Allgemeinbezogene Aspekte**					
*KKT wird im Medizinstudium ausreichend abgedeckt*	Präsenz Online	102 29	2,90 3,21	0,94 1,05	0,135
Besitz ausreichender Kompetenzen gegenüber Patient*innen mit Kommunikationsdefiziten	Präsenz Online	102 29	2,99 3,00	0,80 0,89	0,955
*KKT stellt ein großes Potenzial für die Patientenversorgung dar*	Präsenz Online	102 29	1,77 1,75	0,73 0,95	0,917

*Anzahl n* Zahl der Probanden, *M* Mittelwert, *SD* Standardabweichung, *5‑Punkte-Skala *1 „am besten“ und 5 „am wenigsten“, *KKT* Kommunikative-Kompetenzen-Training, *SP* Simulationspatient*in

### Selbsteinschätzung der Kommunikationskompetenz von Studierenden

Die Selbsteinschätzung der kommunikativen Kompetenz durch die Studierenden finden sich in Tab S1. Nach Berechnung einer Varianzanalyse mit Messwiederholung (ANOVA), in der sowohl die Innersubjekt- als auch die Zwischensubjekteffekte interpretiert wurden, zeigte sich Folgendes: Der Durchschnitt von Zuwachs an kommunikativen Kompetenzen in der Online-Gruppe (M = 1,54 [SD = 0,94]) ist insgesamt doppelt so groß wie in der Präsenzgruppe (M = 0,75 [SD = 0,87]). Die statistische Überprüfung liefert einen signifikanten Unterschied dieser Mittelwerte (*p* < 0,001). Aus der Auswertung der Selbsteinschätzung geht hervor, dass der Zuwachs an kommunikativen Kompetenzen in der Online-Gruppe in allen Fragen mindestens genauso gut war wie in der Präsenzgruppe. In 8 Fragen war die mittlere Verbesserung sogar signifikant größer als in der Präsenzgruppe (Tab. S1). In den Fragen 5 (Blickkontakt) und 7 (angemessene Pausen) hat die Präsenzgruppe sich kaum verbessert.

Außerdem wurde die Möglichkeit gegeben, freie Kommentare abzugeben. Die Analyse der Kommentare von Studierenden im Online-Kurs zeigte, dass das Online-Format insgesamt eine hohe Akzeptanz erzielen konnte. Studierende hielten besonders gern den Kontakt zu Mitstudent*innen und Tutor*innen aufrecht, während die meisten Lernaktivitäten pandemiebedingt ausfielen. Der weggefallene Anfahrtsweg war ein weiterer Vorteil. Es herrschte eine entspannte Atmosphäre, und die Studierenden waren weniger gehemmt, Feedback zu geben. Ebenfalls ermöglichte das Online-Format ein Üben für die Telemedizin, was als wichtig erachtet wurde. Kritik gab es v. a. bezüglich technischer Störungen. Darüber hinaus wurde vermerkt, dass die nonverbalen Aspekte der Kommunikation deutlich schwieriger zu evaluieren waren. Die Studierenden wünschten sich außerdem mehr Inhalte zur Gebärdensprache und mehr klinische Informationen zur Diagnose und Therapie der Schwerhörigkeit.

## Diskussion

Die Vermittlung kommunikativer Fertigkeiten in der HNO ist nach Wissen der Autoren eher selten in der medizinischen Lehre zu finden. Pandemiebedingt musste das Seminar in ein Online-Format überführt werden. Im Rahmen der vorliegenden Arbeit interessierte dabei, ob das Seminar gleich gut oder besser evaluiert werden würde und ob die Vermittlung kommunikativer Fertigkeiten auch online machbar sein könnte. Beide Seminare wurden gut evaluiert. Die Selbsteinschätzung der Studierenden in Bezug auf ihre kommunikativen Fertigkeiten war im Online-Kurs sogar höher als im Präsenzkurs.

Diese Befunde stimmen mit denen ähnlicher Studien über Online-Formate überein [13, 16, 17]. Von besonderer Bedeutung ist hierbei, dass die Studierenden von einer höheren Selbsteinschätzung in den angestrebten Kommunikationsfertigkeiten im Anschluss an das Online-Modul berichtet haben. Die vorliegende Studie unterstreicht die Ergebnisse von Cantone et al. und Junod Perron et al. [12, 21], dass der Einsatz von Online-SP eine gute Erfahrung sein kann, Studierende auf Gespräche online vorzubereiten. Dieses Ergebnis ist auch vereinbar mit den Resultaten von Pei u. Wu [22], bei denen ein signifikanter Unterschied zwischen den Online- und Offline-Lerngruppen zugunsten des Online-Lernens bestand.

Die gute Bewertung des Feedback-Teils ist vereinbar mit den Resultaten von Newcomb et al. [17]. Die Studierenden haben berichtet, dass das Üben von Fertigkeiten mit SP, welches eine direkte Beobachtung der Fähigkeiten ermöglicht, verbunden mit sofortigem Feedback den größten Einfluss auf ihr Lernen hatte.

## Limitationen

Die größte Limitation der vorliegenden Studie ist, dass sie nicht in einem kontrollierten randomisierten Design durchgeführt wurde. Der Online-Kurs und der Kurs vor Ort waren zwar weitgehend vergleichbar. Aber dennoch ist nicht bekannt, wie die Zeit der Pandemie und der Aspekt der freien Teilnahme, die Gruppengröße und die Zusammensetzung des 3. und 4. klinischen Semesters die Ergebnisse beeinflusst hat. Die Ergebnisse sollten daher in einer randomisierten kontrollierten Studie repliziert werden, um die vorliegenden Ergebnisse zu validieren.

In den Seminaren wurde die Evaluation und die Selbsteinschätzung der Studierenden erfasst. Es wurden keine objektiven Daten erhoben, wie z. B. die aktuelle Gesprächsführung, die dann über unabhängige Rater hätte evaluiert werden können. In einer aufwendigeren Studie könnte dies realisiert und dann validierte Rückschüsse auf die kommunikative Kompetenz gezogen werden. Dennoch interpretieren die Autoren ihre Ergebnisse als Hinweise, dass die kommunikative Kompetenz auch im Online-Format gesteigert werden kann.

Weitere Forschungsbemühungen, um die kommunikativen Kompetenzen der Medizinstudierenden als angehende Ärzt*innen zu fördern und diese an verschiedene Patientenklientele und Rahmen wie Telemedizin zu adaptieren, sind erforderlich. Auch die verschiedenen Online-Hilfsmittel, die für Schwerhörige in einem virtuellen ärztlichen Gespräch hilfreich sein können, konnten in dieser Studie nicht explizit erforscht werden, würden jedoch eine umfangreichere Perspektive auf die telemedizinische Versorgung eröffnen.

## Fazit für die Praxis

- Kommunikationstrainings im Bereich der HNO-Heilkunde werden gut evaluiert und können die Selbsteinschätzung der Studierenden verbessern.
- Ein Kommunikationstraining kann auch online durchgeführt werden.
- Kommunikative Fertigkeiten sollten longitudinal in verschiedene klinische Fächer integriert werden.
- Auch nach der Pandemie sollte im Fokus stehen, Medizinstudierende auf telemedizinische Angebote vorzubereiten.

### Supplementary Information





## Abkürzungen

ANOVA: Varianzanalyse
HNO-Heilkunde: Hals‑, Nasen- und Ohrenheilkunde
KKT: Kommunikative-Kompetenzen-Training
M: „Mean“, Mittelwert
*p*: Signifikanz
SD: „Standard deviation“, Standardabweichung
SP: „Standardized patients“, Simulationspatient*in
VAS: „Visual analogue scale“, visuelle Analogskala
