# Supplementary material for: Communication training in otorhinolaryngology education: Comparison of an online and a classroom-based training course
Source: HNO. 2024 Apr 10;72(5):334–40. [Article in German] doi: 10.1007/s00106-024-01475-2 (PMC11045617; doi:10.1007/s00106-024-01475-2)
Supplement: Supplementary file 1 [file 106_2024_1475_MOESM1_ESM.pdf]

Tabelle S1 Selbsteinschätzung zu kommunikativen Kompetenzen vor und nach dem Kurs

	N (P)	t1 (M) (P)	t1 (SD) (P)	t2 (M) (P)	t2 (SD) (P)	Δt1-t2 (M) (P)	Δt1-t2 (SD) (P)	N (O)	t1 (M) (O)	t1 (SD) (O)	t2 (M) (O)	t2 (SD) (O)	Δt1-t2 (M) (O)	Δt1-t2 (SD) (O)	*p*
**1. Kontaktaufnahme**	108	3.35	1.86	2.82	1.50	0.53	1.76	25	3.95	2.31	2.31	1.39	1.64	2.12	0.007
**2. Selbstbewusstsein**	108	4.33	1.66	3.65	1.80	0.68	1.57	25	4.18	2.06	2.55	1.28	1.63	1.98	0.010
**3. Klare Strukturierung von Gesprächen**	108	2.72	1.54	2.36	1.45	0.36	1.41	25	4.07	1.62	2.39	1.45	1.68	1.51	<0.001
**4. Angemessene Sprache**	108	3.76	1.64	3.01	1.61	0.75	1.53	25	3.30	1.58	2.14	1.45	1.16	1.69	0.240
**5. Blickkontakt**	108	3.43	1.46	3.39	1.68	0.04	1.69	25	2.61	1.62	1.46	1.13	1.14	1.57	0.004
**6. Zugewandte Körperhaltung**	108	2.18	1.73	1.84	1.51	0.35	1.67	25	2.31	1.33	1.54	0.77	0.77	1.38	0.239
**7. Angemessene Pausen**	108	2.01	1.73	1.98	1.59	0.03	1.66	25	3.48	1.94	1.97	1.18	1.52	1.53	<0.001
**8. Kurze Sätze**	108	4.07	1.81	3.18	1.67	0.89	1.69	25	3.57	1.71	2.26	1.14	1.32	1.64	0.260
**9. Angemessene Verwendung von Hilfsmitteln**	108	4.63	1.92	3.56	1.81	1.07	1.93	25	3.97	1.89	2.44	1.85	1.53	1.80	0.276
**10. Verständnis durch Patient*innen**	108	4.12	1.89	2.91	1.60	1.21	1.83	25	3.55	1.40	2.25	1.67	1.30	1.33	0.821
**11. Angemessener Gesprächsabschluss**	108	4.11	1.71	3.02	1.44	1.09	1.68	25	3.57	1.64	2.38	1.54	1.19	1.34	0.789

**12. Erkennen der Emotionen der Patient*innen**	108	4.11	1.92	3.50	1.82	0.61	2.04	25	2.85	1.45	2.14	1.65	0.71	1.66	0.818
**13. Emotionale Unterstützung der Patient*innen**	108	3.90	1.31	3.52	1.41	0.38	1.15	25	3.63	1.69	2.26	1.23	1.37	1.85	0.016
**14. Qualität des Gesprächs**	108	4.00	1.66	3.62	1.61	0.38	1.74	25	4.02	1.42	2.48	0.94	1.54	1.34	0.002
**15. Theoretische Kenntnisse über Gespräche in HNO-Heilkunde**	108	5.73	1.75	3.38	1.69	2.35	1.95	25	5.70	2.99	2.30	1.71	3.40	2.89	0.094
**16. Anwendung theoretischen Wissens**	108	5.02	1.70	3.72	1.60	1.30	1.62	25	5.08	2.34	2.35	1.51	2.73	2.14	<0.001

FR=Fragenummer, N= Zahl der Probanden, M= Mittelwert, SD= Standardabweichung, *p*= Signifikanz Mittelwerte, P=Präsenz-Gruppe, O=Online-Gruppe, T1= prä-Befragung, T2= post-Befragung, ΔT1-T2= Differenz Selbsteinschätzung, 10-Punkte-Skala: 1 „sehr sicher“ und 10 „sehr unsicher“
